# Expression of non-neuronal cholinergic system in maxilla of rat *in vivo*

**DOI:** 10.1186/0717-6287-47-72

**Published:** 2014-12-17

**Authors:** Jie Guo, Lue Wang, Haihua Xu, Xiaoxia Che

**Affiliations:** Department of Orthodontics, School of Stomatology, Shandong University; Shandong Provincial Key Laboratory of Oral Biomedicine, Jinan, 250012 People’s Republic of China; College of Life Science and Technology, Beijing University of Chemical Technology, Beijing, 100029 People’s Republic of China; Department of Orthodontics, School of Stomatology, Capital Medical University, No. 4 Tiantan Xili, Dongcheng District, Beijing, 100050 People’s Republic of China

**Keywords:** Non-neuronal cholinergic system, Maxilla, Real-Time PCR, Immunohistochemistry

## Abstract

**Background:**

Acetylcholine (ACh) is known to be a key neurotransmitter in the central and peripheral nervous systems, which is also produced in a variety of non-neuronal tissues and cell. The existence of ACh in maxilla *in vivo* and potential regulation role for osteogenesis need further study.

**Results:**

Components of the cholinergic system (ACh, esterase, choline acetyltransferase, high-affinity choline uptake, n- and mAChRs) were determined in maxilla of rat *in vivo*, by means of Real-Time PCR and immunohistochemistry. Results showed RNA for CarAT, carnitine/acylcarnitine translocase member 20 (Slc25a20), VAChT, OCTN2, OCT1, OCT3, organic cation transporter member 4 (Slc22a4), AChE, BChE, nAChR subunits α1, α2, α3, α5, α7, α10, β1, β2, β4, γ and mAChR subunits M1, M2, M3, M4, M5 were detected in rat’s maxilla. RNA of VAChT, AChE, nAChR subunits α2, β1, β4 and mAChR subunits M4 had abundant expression (2^-ΔCt^ > 0.03). Immunohistochemical staining was conducted for ACh, VAChT, nAChRα7 and AChE. ACh was expressed in mesenchymal cells, chondroblast, bone and cartilage matrix and bone marrow cells, The VAChT expression was very extensively while ACh receptor α7 was strongly expressed in newly formed bone matrix of endochondral and bone marrow ossification, AchE was found only in mesenchymal stem cells, cartilage and bone marrow cells.

**Conclusions:**

ACh might exert its effect on the endochondral and bone marrow ossification, and bone matrix mineralization in maxilla.

**Electronic supplementary material:**

The online version of this article (doi:10.1186/0717-6287-47-72) contains supplementary material, which is available to authorized users.

## Background

Acetylcholine (Ach) acting solely as neurotransmitter has been revised by findings published both early and late in the last century which all demonstrated the existence of Ach in non-neuronal systems [1, 2]. Cholinergic communication and regulation are established from the beginning of life, that is, in primitive uni- and multicellular organisms such as bacteria, algae, protozoa, sponge and primitive plants and fungi [3–5].

Increasing evidence indicates that ACh acts as an paracrine and autocrine signaling molecule which controlling basic cell functions, such as proliferation, dif ferentiation, cell–cell contact, immune functions, secretion, and absorption in non-neuronal cell including epithelial, endothelial, mesothelial, immunocompetent, and smooth muscle cells [2, 6, 7]. Non-neuronal cells possess cholinergic components uptake choline by means of the high affinity choline transporter (CHT1) [8], and then synthesize ACh by choline acetyltransferase (ChAT) [9] from choline and acetyl-coenzyme A (acetyl-CoA).

ACh is translocated into small synaptic vesicles by vesicular ACh transporter (VAChT), and released via exocytosis [10]. Once released, ACh exerts its cellular functions via nicotinic acetylcholine receptors (nAChRs), including 16 subunits (α1, α2, α3, α4, α5, α6, α7, α9, α10, β1, β2, β3, β4, γ, δ and ϵ) and muscarinic acetylcholine receptor (mAChRs), which including 5 subtypes (M1, M2, M3, M4 and M5). Finally, ACh is rapidly degraded into choline and acetate by acetylcholinesterase and butyrylcholinesterase (AChE and BChE) [11].

As described in the non-neuronal cholinergic systems of other cell types and tissues, the presence of all the necessary molecular components of ACh synthesis, release, reception, degradation, and reuptake existed in mouse and human osteoblast-like cells [12]. Osteoblasts express specific acetylcholine receptors and cholinergic components, and ACh plays a potential role in regulating the proliferation and differentiation of osteoblasts [13]. nAChRs include the α2 nAChRs subunit have a key role in regulating skeletal remodeling mainly through an inhibitory tone of bone resorption [14]. However, whether Ach existed in bone *in vivo* is still undetermined. In this study, we studied the existence of ACh in maxilla *in vivo* and their potential regulation role for osteogenesis.

## Results and discussion

### The morphology of maxilla

**Figure 1:**
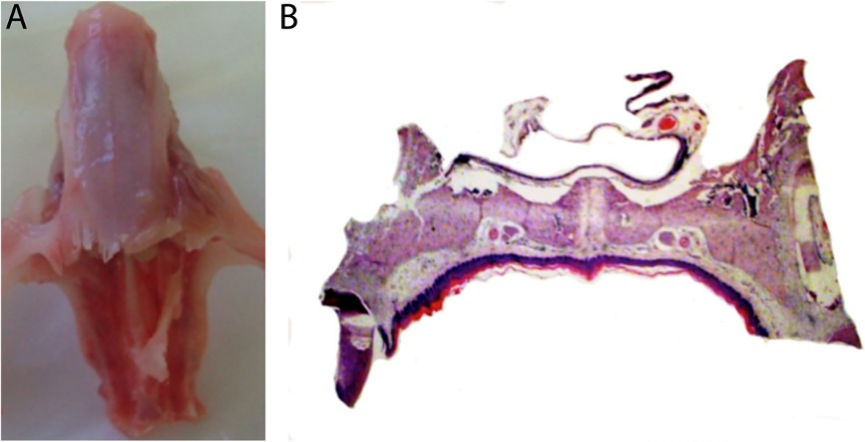
**Maxilla from a normal SD rat: A, Maxilla specimen for PCR analysis; B, The palatal shelf structure of maxilla.**

Essentially, mammalian bones are in the form of 2 different ways: long bones via endochondral ossification; and flat bones via intramembranous ossification. Orofacial bone is mainly formed via intramembranous ossification [15, 16]. These bony types show considerable differences in protein composition [17]. The morphology of maxilla harvested in this study showed in Figure 1A. A complete palatal shelf structure in a normal SD rat is shown in Figure 1B. The bony plates were interposed between two layers of soft tissue, the nasal and oral mucosa. The palatine vessels and nerves formed large bundles and were housed in bilateral concavities in the bone, and a single layer of osteoblasts lined the oral and nasal surfaces of the bone.

### Real-Time PCR analysis of the components of non-neuronal cholinergic system in maxilla

We screened RNA for the expression of all non-neuronal cholinergic system components, including esterase, choline acetyltransferase, high-affinity choline uptake, organic cation transporters, carnitine/acylcarnitine translocase, n- and mAChRs. Result showed CHT1, ChAT, OCT2, SLC25a29 and nAChR subunits α4, α6, α9, β3, δ, ϵ were not detected in maxilla. While the RNA of AChE, BChE, CarAT, carnitine/acylcarnitine translocase member 20 (Slc25a20), VAChT, OCTN2, OCT1, OCT3, organic cation transporter member 4 (Slc22a4), nAChR subunits α1, α2, α3, α5, α7, α10, β1,β2, β4, γ and mAChR subunits M1, M2, M3, M4, M5 were detected in rat’s maxilla.

**Figure 2:**
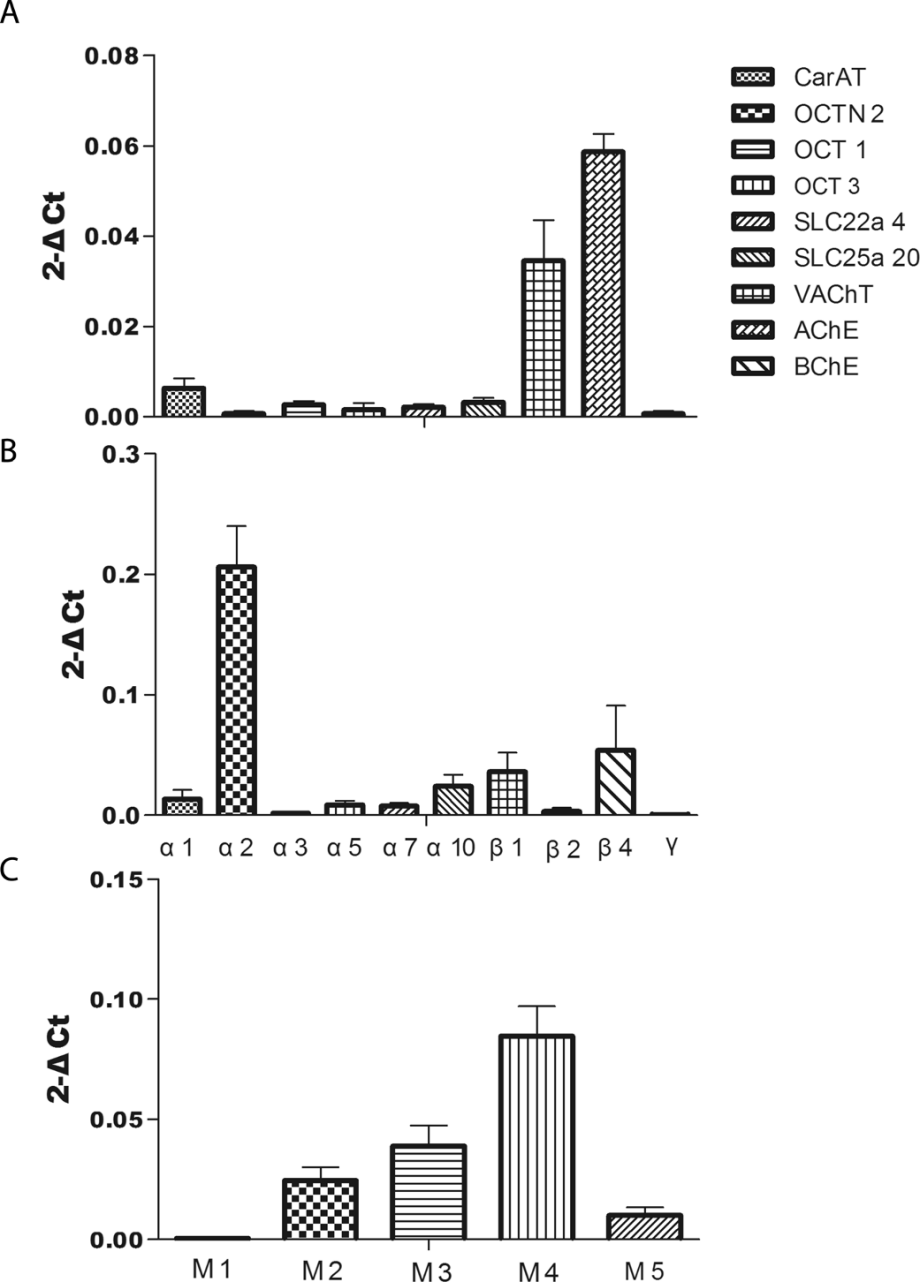
**Gene expression of the non-neuronal cholinergic system: A, The expression of synthesis transport and degradation systems, including CarAT, OCTN2, OCT1, OCT3, SLC22a4, SLC25a20, VAChT, AChE, BChE; B, The expression of nAChR subunits, including α1, α2, α3, α5, α7, α10, β1,β2, β4 and γ; C, The expression of mAChR subunits, including M1, M2, M3, M4 and M5.**

The RNA of VAChT, AChE, nAChR subunits α2, β1, β4 and mAChR subunits M4 had abundant expression (2^-ΔCt^ > 0.03) (Figure 2). And RNA of CarAT, OCT1, nAChR subunits α1, α5, α7, α10, mAChR subunits M2, M3 had less abundant expression(0.005 ≤ 2^-ΔCt^ ≤ 0.03), and the other components had low expression level (2^-ΔCt^ < 0.005).

### Immunohistochemistry of ACh, VAChT, nAChRα7 and AChE in maxilla

Traditional methods for the detection of acetylcholine were high performance liquid chromatography (HPLC) and micro dialysis, but location of acetylcholine could not be determined by these methods. Antibodies of choline-protein conjugates introduced by Lolin et al. provided a potentially method to detect ACh [18]. Later Schlereth et al. found conjugated ACh--glutaryl-BSA (bovine serum albumin) the most immunoreactive immunogen by the injection of different ACh- conjugates into AKR and DBA mice followed by ELISA detection for the affinity and specificity of immune serum [19]. The immunogen of anti-acetylcholine polyclonal antibody used for our immunohistochemistry was also acetylcholine-glutaric anhydride- poly lysine.

**Figure 3:**
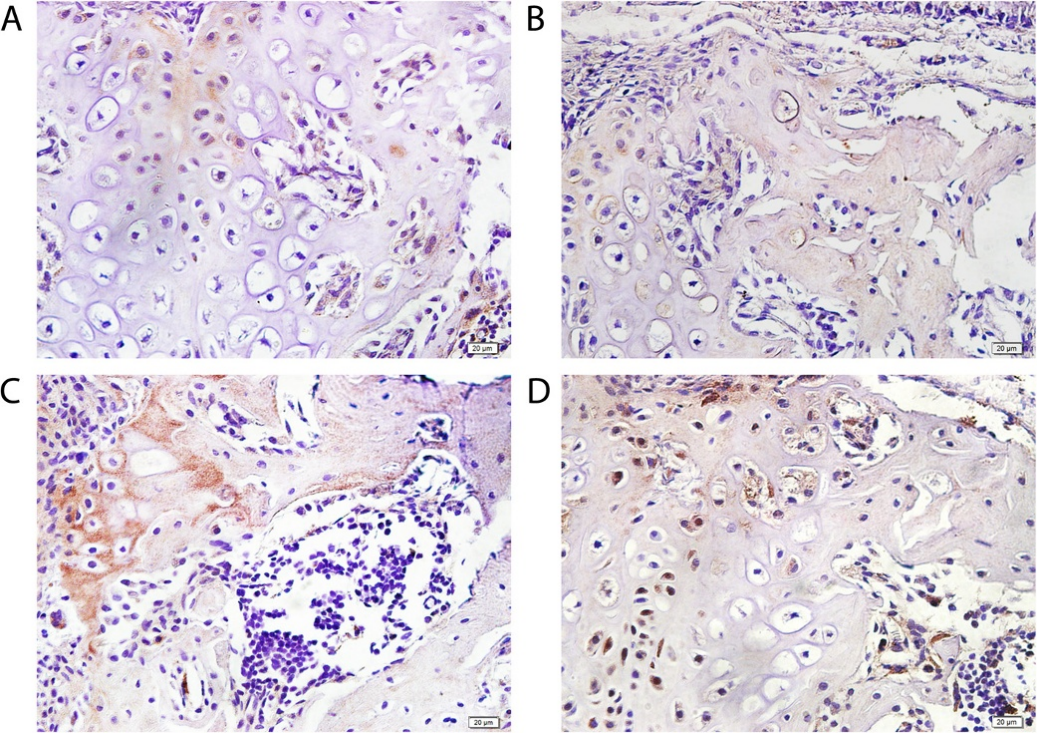
**Immunohistochemical results of ACh, VAChT, α7 and AChE in maxilla cells: A, ACh was expressed in mesenchymal stem cells, chondroblast, bone and cartilage matrix and bone marrow cells; B, VAChT was expressed in mesenchymal stem cells, chondroblast and cartilage; C, nAChRα7 was expressed in bone matrix of endochondral and bone marrow ossification, and bone marrow cells; D, AChE was expressed in mesenchymal stem cells, cartilage and bone marrow cells.**

Figure 3 demonstrated the immunohistochemistry results. As we can see, ACh was expressed in mesenchymal stem cells, chondroblast, bone and cartilage matrix and bone marrow cells (Figure 3A), this indicates that the maxilla also expressed non-neuronal acetylcholine. The immunohistochemistry results of negative control and positive control of ACh antibodies can be found in Additional file 1: Figure S1. The VAChT was expressed very extensively, but less intense (Figure 3B), which showed that ACh was transported to many parts of the maxilla, and played a part in bone metabolism. As ACh receptor, α7 are strongly expressed in newly formed bone matrix of endochondral and bone marrow ossification (Figure 3C), which showed that ACh must be involved in the formation of bone matrix. The AChE, degrading of acetylcholine, had a narrow distribution, only in mesenchymal stem cells, cartilage and bone marrow cells (Figure 3D), which indicats that ACh might contribute to the metabolism in the maxillary bone of normal rats.

ACh was synthesized by practically all living cells and can play an intermediary role in the interactions of non-neuronal cells with the external environment, hormones, growth factors, cytokines and also the neural system [20–23]. Immunohistochemical staining of trabecular bone in the distal femoral metaphysis demonstrated VAChT positive regional in medullary intertrabecular spaces, mainly in the close vicinity of bony struts, one-to-three cell layers away from their surface [14]. The alpha-7 nicotinic acetylcholine receptor (α7-nAChR) was well known as a potent calcium ionophore. Rogers et al. described the expression of α7 during development of the teeth and show that for this nicotinic receptor the distinct spatial and temporal differences in its expression suggested functional pleiotropy in the tooth developmental process [24]. AChE had been detected as nuclear protein in both neuronal and non-neuronal cells [25]. The chondrogenic expression of AChE paralleled the early development of rat lower limbs [26]. AChE fulfilled at least some of the requirements for an osteogenic coordinator.

In maxilla ACh might be produced by carnitine acetyltransferase (CarAT), not choline acetyltransferease (ChAT), which was consistent with some non-neuronal cells such as muscle cells and urothelial epithelial cells [9, 27]. Bajayo et al. [14] demonstrated that VAChT had been expressed in trabecular bone in the distal femoral metaphysic when compared the expression of VAChT mRNA with OCTs’, this indicated that bone transport ACh by VAChT. They also find AChE expressed extremely rich, whereas BChE expressed very little, this illustrated that ACh was degraded mainly by AChE in maxilla.

Sato et al. showed that Nic, an agonist of nAChRs, induced proliferation of osteoblasts [28]. It was demonstrated previously that osteoblasts mainly expressing the muscular type α1, β1, and γ subunits, as well as the neuronal subunits α4, α7, β2, and β4 [14]. But our Real-Time PCR result indicated that many components of nAChRs existed in maxilla, especially the subunits α1, α2, α10, β1 and β4. On the other hand, the ingredients of mAChRs expressed largely, except M1 subunit. Kliemann et al. [29] indicated a significant decrease in relative bone volume, trabecular thickness, trabecular number, bone surface density, and a significant increase in trabecular separation and structure model index of M3R–KO using Micro-CT analysis. All these show that non-neuronal cholinergic system involved in bone formation.

We hypothesized that ACh plays a functional role in bone metabolism. ACh increased the viability, but decreased the proliferation of embryonic stem cells [30]. ACh was expressed in mesenchymal cells, chondroblast, bone and cartilage matrix and bone marrow cells, so we suppose that ACh might participate in the metabolism of the maxilla. ACh had abundant expression in mesenchymal cells, chondroblast and cartilage matrix, this illustrating that ACh might be related with membrane bone and cartilage mineralization. In addition, ACh has been expressed in bone matrix and bone marrow cells, so, we hypothesized that acetylcholine had relevant to bone marrow ossification.

ACh, VAChT, nAChR α7 and AChE are expressed in bone marrow mesenchymal stem cells; ACh might participate in the proliferation, differentiation and death of bone marrow stem cells. ACh, VAChT and nAChR α7 are expressed in bone matrix of endochondral and bone marrow ossification, but AChE is not expressed, further illustrated that the non-neuronal cholinergic system participated in osteogenesis and bone matrix mineralization. These four ingredients were expressed in chondroblast and cartilage cells, indicating that the system were concerned with the cartilage cells formation, and might be involved in cartilage ossification.

## Conclusions

The non-neuronal cholinergic system is expressed in the maxilla. ACh mainly transported by VAChT, degraded by AChE, and came into play by parts of nAChR and mAChR subunits, major α1, α2, α10, β1, β4 and M2, M3, M4, M5. The immunohistochemistry of 4 constituents (ACh, VAChT, nAChRα7 and AChE) indicating that non-neuronal cholinergic system participated in the proliferation, differentiation and death of mesenchymal stem cells, cartilage and bone marrow cells, worked in the endochondral and bone marrow ossification, and bone matrix mineralization as well. However, the role of non-neuronal cholinergic system still need be further researched.

## Methods

### Animals and ethics statement

All of the rats were treated according to the ethical regulations defined by the Ethics Committee of Capital Medical University.

Male Sprague–Dawley rats purchased from Research Institute of Drug Inspection of China (n = 9, 7–8 week-old, mean weight of 302.14 ± 8.42 g). All rat were decapitated under anesthesia on 7th day, and the maxilla was harvested (Figure 1A). Specimens from 6 rats were used for Real-Time PCR analysis and specimens from the other 3 rats were used for immunohistochemistry analysis.

### Real-Time PCR analysis of the components of non-neuronal cholinergic system in maxilla

Real-Time PCR analysis was performed with a Roche 480 Sequence Detection System according to manufacturer’s instructions (ROCHE GROUP, Basel, Switzerland) for validation of gene expression of the cholinergic system.

The maxilla from the first to the third molars in rats were harvested free of the overlying soft tissue. Specimens were ground under liquid nitrogen, and then mixed with Trizol solution (Invitrogen, Carlsbad, CA, USA). RNA was treated with DNase I (TAKARA, DALIAN) to eliminate genomic DNA. The yield and purity of RNA was estimated spectrophotometrically using the A260/A280 ratio and agarose gel electrophoresis. RNA was reverse transcribed to cDNA using the superscript III reverse transcriptase (Invitrogen, Carlsbad, CA, USA) according to the manufacturer’s instructions.

The cDNAs were amplified with gene-specific primer pairs (listed in Table 1). Reactions were run on the Roche 480 Real-Time PCR detection system and the results were analyzed using the software supplied with the machine. The running conditions were: incubated at 50°C for 2 minutes and 95°C for 5 minutes, followed by 45 cycles of incubation at 95°C for 15 s and 60°C for 1 minute. The β-actin of rat and 18S gene was used as 2 internal controls. The expression levels of the target genes were correlated to mean of β-actin and 18 s using the 2^-ΔCt^ method.

**Table 1:** **Sequences and accession numbers for forward and reverse primers used in Real-Time PCR**

Name	Genbank No.	Forward	Reverse
CarAT	NM_001004085	ATTGTCGCTCTTGTGGACC	TCTGTTTGGCCTTCTCTATGTC
OCTN2	NM_019269	CGATCCCAGTGAGTTACAAGAC	GAGAAAGTCCGAAGTAGCCC
OCT1	NM_012697	TGGCCGTAAGCTCTGTCTCT	TCAAGGTATAGCCGGACACC
OCT3	NM_019230	CAATGGGAAACACCTCTCGT	ATACACCACGGCACTTGTGA
SLC22a4	NM_022270	CTGGGAGTACAGCAAGGA	GAAGGAGCCACAGAGAAC
SLC25a20	NM_053965	AACCCATCAGTCCGCTTAAG	TGGTCCCAGAGTACATAGGTG
VAChT	X80395	GCCACATCGTTCACTCTCTTG	CGGTTCATCAAGCAACACATC
AChE	S50879	CTTCTCCCACACCTGTCC	CTGCTTCCTGGTAGAGCC
BChE	NM_022942	ACCTAACCTTGAACACAGAGAAG	TTCCACTCTTGCTCCCTTTC
α1	NM_024485	GTCACCCACTTTCCCTTCGA	CAGGTCGGGCTGGTCACTT
α2	NM_133420	CGCTGGTCATCCCACTCAT	GGGAGCGGTGGTGTACATTG
α3	NM_052805	TCCAGTTTGAGGTGTCCATG	CTTGGTAGTCAGAGGGTTTCC
α4	NM_024354.1	CGCATCCCCTCTGAACTCAT	CACTGCACCCTTCCGTCATA
α5	NM_017078	TGGACGCAACCAGCAAACTA	TATGTCCACGAGCCGAATTTC
α7	AY574256	GCTGGTTCCCTTTTGATG	CTCCGTTGGGGATATAGC
α10	AF196344	CAGTCTCTCCCCAAAGTG	GAGGTGGGCTTTAGATCC
β1	X74833	TGGTTGTGGACCGTCTTTTTC	ATGACCGGAGGGTCCTCAAG
β2	L31622	TTCCTGCTGCTCATCTCCAA	CGCTGGTGACGATGGAGAA
β3	NM_133597.1	TGGAAACACTCTGCGCTTGA	TCCTGCAGTGGCCGTAAGA
β4	AY574260	TGCTGGCACTCACGTTCTTC	AAGGTGACCAGCACCATGGT
γ	NM_019145	CACCTACTTCCCCTTCGATTG	ATTCTCTGTGAAAGCCTCGG
M1	NM_080773	TGGTTTCCTTCGTTCTCTGG	GAGGAACTGGATGTAGCACTG
M2	J03025	CCACTCCAGAGATGACAACT	GGCTACAACGTTCTGCTTT
M3	M16407	GGACTGTGGATGTGGAGAG	CGAGGAGTTGGTGTCAGA
M4	NM_031547	CCCGCCGCACTACTAAGATG	CCTCTTGCCCACCACAAACT
M5	M22926	CAGCTGCTGCTCACAGACTCA	GGGAAGGAACAGGGCATGAT
β-actin	NM_031144	CTTCAACACCCCAGCCATGT	CAGAGGCATACAGGGACAACA
18S	M11188	GCGGTTCTATTTTGTTGG	AATGCTTTCGCTCTGGTC

### Hematoxylin and eosin staining of maxilla

The palates from the first to third molars of rats were fixed in 10% formalin solution for 48 h. The blocked tissues were demineralized in 10% (w/v) ethylenediaminetetra-acetic acid (EDTA) for 14 days at 4°C. Routine paraffin embedding procedures were performed. Sections (5 μm thick) were cut and mounted on poly-L-lysine–coated glass slides. Before staining, sections were incubated at 60°C for 1 h and then held in xylene and rehydrated through a series of ethanol solutions. Sections were stained with hematoxylin and eosin to observe the histological morphology. The staining imaging was captured using an OLYMPUS BX61 (Tokyo, Japan) optical microscope.

### Immunohistochemistry of Ach, VAChT, nAChR α7 and AChE

Prepared sections were reacted with anti-ACh (Chemicon), anti-VAChT (Abcam), anti-α7 nAChR (Abcam) and anti-AChE (Novus) antibodies. An immunohistochemical staining secondary antibodies kit (Maixin Biotechnology, Inc., Fuzhou China) was used according to the manufacturer’s instructions, and then colored with DAB (Maixin Biotechnology, Inc., Fuzhou China). Sections were counterstained with hematoxylin before they were covered. Immunohistochemical staining imaging was captured using an OLYMPUS BX61 (Tokyo, Japan) optical microscope.

### Statistical analysis

Data were expressed as mean ± standard deviation. The data was subjected to ANOVA analysis. P < 0.05 were considered as significant. Statistical analyses were performed using the SPSS 17.0 software.

## Electronic supplementary material

Additional file 1: Figure S1: **Negative control and positive control of ACh antibodies.** (TIFF 3 MB)
