# Supplementary figures and images for: Expression of non-neuronal cholinergic system in maxilla of rat in vivo
Source: Biol Res. 2014 Dec 17;47(1):72. doi: 10.1186/0717-6287-47-72 (PMC4289578; doi:10.1186/0717-6287-47-72)

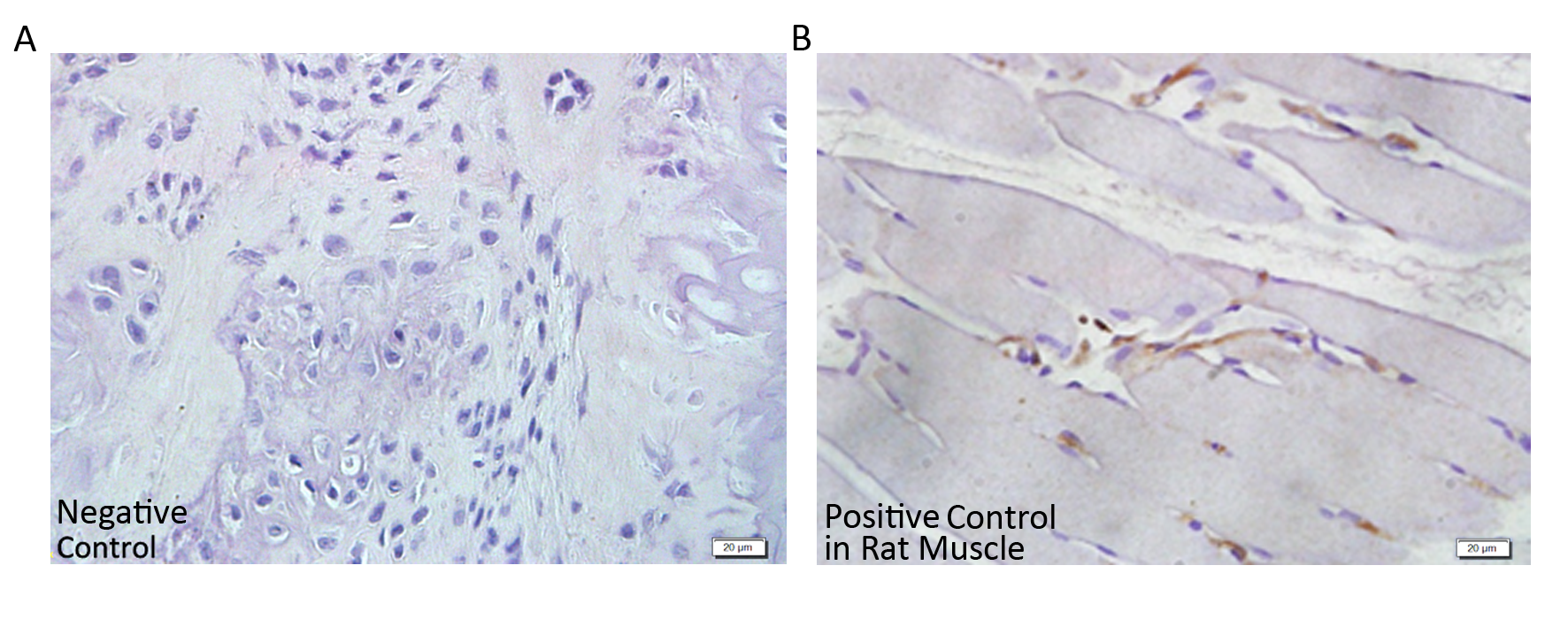

Supplement: Supplementary file 1 — Additional file 1: Figure S1: Negative control and positive control of ACh antibodies. (TIFF 3 MB) [file 40659_2014_59_MOESM1_ESM.tiff]
